# The Broad Spectrum of *LMNA* Cardiac Diseases: From Molecular Mechanisms to Clinical Phenotype

**DOI:** 10.3389/fphys.2020.00761

**Published:** 2020-07-03

**Authors:** Silvia Crasto, Ilaria My, Elisa Di Pasquale

**Affiliations:** ^1^Humanitas Clinical and Research Center – IRCCS, Rozzano, Italy; ^2^Institute of Genetic and Biomedical Research (IRGB) – UOS of Milan, National Research Council (CNR), Milan, Italy

**Keywords:** *LMNA* gene, cardiolaminopathy, Lamin A/C, clinical phenotype, molecular mechanisms

## Abstract

Mutations of Lamin A/C gene (*LMNA*) cause laminopathies, a group of disorders associated with a wide spectrum of clinically distinct phenotypes, affecting different tissues and organs. Heart involvement is frequent and leads to cardiolaminopathy LMNA-dependent cardiomyopathy (LMNA-CMP), a form of dilated cardiomyopathy (DCM) typically associated with conduction disorders and arrhythmias, that can manifest either as an isolated event or as part of a multisystem phenotype. Despite the recent clinical and molecular developments in the field, there is still lack of knowledge linking specific *LMNA* gene mutations to the distinct clinical manifestations. Indeed, the severity and progression of the disease have marked interindividual variability, even amongst members of the same family. Studies conducted so far have described Lamin A/C proteins involved in diverse biological processes, that span from a structural role in the nucleus to the regulation of response to mechanical stress and gene expression, proposing various mechanistic hypotheses. However, none of those is *per se* able to fully justify functional and clinical phenotypes of LMNA-CMP; therefore, the role of Lamin A/C in cardiac pathophysiology still represents an open question. In this review we provide an update on the state-of-the-art studies on cardiolaminopathy, in the attempt to draw a line connecting molecular mechanisms to clinical manifestations. While investigators in this field still wonder about a clear genotype/phenotype correlation in LMNA-CMP, our intent here is to recapitulate common mechanistic hypotheses that link different mutations to similar clinical presentations.

## Introduction

### *LMNA* Gene and Its Products

*LMNA* gene maps to chromosome 1q21.1-21.2 and is composed of 12 exons spanning around 25 kb. It encodes A-type nuclear lamins via alternative splicing (Lin and Worman, 1993; Wydner et al., 1996): Lamin A and C (Lamin A/C) represents the two main isoforms, while Lamin C2 and AD10 are described in germ cells and cancer cells, respectively (Machiels et al., 1996; Alsheimer et al., 2000). A-type lamin proteins have a tripartite domain organization with a central rod domain, a short N-terminal head domain and a tail C-terminal domain. Unlike Lamin C, Lamin A is translated as Prelamin A, containing a carboxyl-terminal CaaX motif, which is then modified by carboxymethylation and farnesylation and undergoes sequential post-translational modifications to form mature Lamin A (Herrmann and Aebi, 2004; Young et al., 2005). Nuclear lamins are classified as type V intermediate filament (IF) proteins and represent the major elements that constitute the nuclear lamina (NL). In addition to their well-known structural role in the nucleus, Lamin A/C are increasingly considered as key players in regulating gene transcription through both direct and indirect modulation of chromatin organization, DNA replication and signal transduction pathways (Gruenbaum et al., 2005; Dechat et al., 2008; Dittmer and Misteli, 2011).

Unlike B-type lamins, the expression of A-type lamins is developmentally regulated and mostly occurs in differentiated cells (Worman and Bonne, 2007). During mouse embryogenesis, Lamin A/C start to be expressed around day 8–9 in extra-embryonic tissues and few days later (day 10–12) in the embryo (Dittmer and Misteli, 2011). The cell-specific and temporally regulated expression of these proteins supports a fundamental role of Lamin A/C in cell differentiation, lineage specification and tissue development (Worman and Bonne, 2007; Butin-Israeli et al., 2012).

### Laminopathies

Mutations in the *LMNA* gene cause laminopathies, a group of disorders characterized by phenotypically heterogeneous manifestations. Up to now a total of 498 *LMNA* mutations have been described^1^ associated to more than 15 different phenotypes. Indeed, laminopathies can either specifically affect distinct tissues, including striated muscles, the peripheral nerves, or the adipose tissue, or present as a systemic disease affecting concomitantly several organs similar to premature aging syndromes. However, there is growing evidence of overlapping phenotypes, suggesting the presence of a real continuum within the disease.

Among the different phenotypes, cardiac involvement is one of the most prevalent and severe manifestations, being a hallmark of several laminopathies, such as Emery-Dreifuss muscular dystrophy, Limb-girdle muscular dystrophy 1B and Hutchinson-Gilford Progeria Syndrome (HGPS) (Fatkin et al., 1999; Arbustini et al., 2002; van Tintelen et al., 2007a). Despite the recent developments in the field, the understanding of the pathogenetic role of Lamin A/C in cardiac disease is still incomplete.

In this review we aim to provide an update on the state-of-the-art investigations in this area of research, drawing a definite line from the molecular mechanisms toward the clinical manifestations. Although investigators are still working on defining genotype/phenotype correlations, our goal is to highlight common molecular features that could link different *LMNA* variants to a similar clinical presentation.

## From *Lmna* Variants to Cardiac Phenotypes

### *LMNA* Variants

The first reports that associate *LMNA* mutations to cardiac diseases date back to 1999 (Bonne et al., 1999; Fatkin et al., 1999): these reports represented a revolution in the field, identifying for the first time *LMNA* mutations as responsible for dilated cardiomyopathy (DCM) associated with conduction system disease. Jakobs et al. (2001) described several novel *LMNA* mutations, one missense (p.E203K, nucleotide c.G607A) in 14 individuals and one nonsense (p.R225X, nucleotide c.C673T) in 10 subjects: these patients were diagnosed with DCM and variable conduction system disease, and were free from any skeletal muscle phenotype. Few years later, other mutations in *LMNA* gene were described (van Tintelen et al., 2007a,van Tintelen et al., 2007b; Saga et al., 2009): one nonsense mutation (815_818delinsCCAGAC) and some missense variants (p.N195K; p.Y259H; p.R166P). Overtime, *LMNA* gene emerged as the second most commonly mutated gene associated to familial cardiomyopathy (CMP), accounting for ∼6–8% of the cases (Hershberger and Siegfried, 2011) of idiopathic DCM, with this number raising up to 33% for cases presenting with both DCM and conduction defects (McNally and Mestroni, 2017).

### Cardiolaminopathy

Cardiomyopathy caused by mutations in *LMNA* gene is referred to as cardiolaminopathy (LMNA-CMP). The disease is generally characterized by variable extent of ventricular dilation (Bonne et al., 1999; Fatkin et al., 1999) or, less frequently, by left ventricular (LV) non-compaction (Sedaghat-Hamedani et al., 2017).

LMNA-CMP has been linked to 165 unique mutations, distributed along the entire gene (Tesson et al., 2014). However, the majority of the mutations occur in the head and in the rod domains and rarely in the tail domain (Fatkin et al., 1999; Jakobs et al., 2001). Pathogenic variants are mainly missense and nonsense mutations, while fewer small deletions/insertions have been identified (Dittmer et al., 2014; Zahr and Jaalouk, 2018). Haploinsufficiency has been suggested as mechanism of disease in patients carrying truncating variants, while missense mutations have been proposed to act mainly through a dominant negative pathway. Subjects with truncating mutations have been associated to an earlier onset of cardiac conduction defects and atrial arrhythmias and a lower LV ejection fraction (EF), than those with missense mutations (Nishiuchi et al., 2017). However, so far there is still lack of knowledge explaining the link between specific *LMNA* mutations and a defined phenotype, and the severity and the progression of the disease have marked interindividual variability, not only among unrelated probands, but also within members of the same family. This supports the concept that the final phenotype results not only from the single *LMNA* mutation, but is also influenced by modifying genes or environmental cues, similarly to what has been reported in some families with titin and desmin mutations (Muntoni et al., 2008; Granger et al., 2011; Roncarati et al., 2013). Whole-exome and whole-genome sequencing studies will unequivocally facilitate the investigation of the genetics behind the disease and allow a comprehensive understanding of the mechanisms underlying the final clinical presentation.

### Cardiac Conduction System Disease and Arrhythmias

The clinical course of LMNA-CMP is characterized by a high rate of major cardiac events such as sudden cardiac death (SCD), malignant ventricular tachycardia (VT), extreme bradycardia due to a high degree of atrioventricular block (AVB) and end-stage heart failure. What is intriguing is the common finding that laminopathies often manifest as primary arrhythmia. Bradyarrhythmias and supraventricular tachyarrhythmias as atrial fibrillation and flutter often anticipate by decades the development of DCM. For this reason, genetic screening should be considered in young patients presenting with new AVB or atypical atrial arrhythmias, even in the absence of LV dysfunction. In the study by Kumar et al. (2016), only one-half of patients have an LVEF < 50% at the initial medical consultation.

The pathophysiological mechanisms underlying the arrhythmic phenotype are still not well elucidated. Systolic dysfunction, male sex, non-missense mutations and non-sustained VT are considered predictors of malignant ventricular arrhythmias in LMNA-CMP (Van Rijsingen et al., 2012; Hasselberg et al., 2014; Kumar et al., 2016; Nishiuchi et al., 2017). However, such risk factors are debated in some studies (Nishiuchi et al., 2017; Peretto et al., 2019) and it was recently shown in a large cohort of DCM patients, that carriers of *LMNA* variants experience the highest rates of SCD/VT/ventricular fibrillation (VF), which was independent of the LV EF (Gigli et al., 2019). Further studies need to be conducted in order to clarify to which extent phenotypic differences among the different cohorts of patients are dependent on genetic background rather than on specific *LMNA* variants.

### Clinical Presentation

The presentation of DCM does not show specific characteristics and the expression of the DCM pattern was found to be age-dependent, with development of the phenotype between 20 and 39 years of age in two thirds of the cases and complete penetrance by 60 years (Arbustini et al., 2002). The “red flags” predicting higher chance of *LMNA* mutations are the concomitant presence of conduction defects and skeletal muscle involvement, even if creatine phosphokinase (CPK – serum marker of muscular damage) is elevated only in one third of the cases (Fatkin et al., 1999; Rapezzi et al., 2013). Arbustini et al. (2002) showed that approximately 33% of patients with AVB and cardiomyopathy carries a *LMNA* mutation. For this reason, screening for *LMNA* mutations in young patients with idiopathic DCM, especially when it is associated with atrial arrhythmias and/or AVB, is important for prognosis and genetic counseling.

Patients who have both cardiac and neuromuscular manifestations more commonly experience bradyarrhythmias and atrial fibrillation compared to patients with solely cardiac phenotypes, despite having no differences in structural heart disease. In this group of patients cardiac abnormalities do not strictly correlate with the severity of the neuromuscular involvement, which may suggest distinct pathogenetic mechanisms (Benedetti et al., 2007; Hasselberg et al., 2018; Peretto et al., 2019). However, a recent study on a small cohort of patients showed that the neuromuscular presentation was associated with earlier cardiac involvement, characterized by a linear and progressive evolution from rhythm disorders to cardiomyopathy (Ditaranto et al., 2019).

### Prognosis and Risk Stratification

Cardiolaminopathy often presents an aggressive and rapid evolution, with a worse natural history compared to other forms of non-ischemic dilated cardiomyopathies and have higher prevalence of malignant arrhythmias and cardiac transplantation (Pasotti et al., 2008; Kayvanpour et al., 2017).

Positive genetic testing for *LMNA* mutations has crucial clinical and prognostic implications. Mortality in patients with LMNA-CMP is estimated to be 40% at 5 years (Pasotti et al., 2008), whereas 45% suffered SCD or aborted SCD. Currently, *LMNA* mutations represent the only genetic background in DCM that influences international guidelines-based timing of ICD therapy in primary prevention, regardless of LV EF values (Priori et al., 2013; Priori and Blomström-Lundqvist, 2015).

Recently, a new 5-year prediction model for life threatening ventricular tachyarrhythmia (LTVTA) has been proposed (Wahbi et al., 2019) to assist us in deciding whether or not a candidate is eligible for the placement of an ICD implantation (online calculator available at http://lmna-risk-vta.fr/). Predictors of LTVTA in the analyzed sample were male sex, non-missense *LMNA* mutation, 1st degree and higher AV block, non-sustained VT and LV EF. The risk threshold used enabled reclassification of 28.8% of patients compared with the guidelines-based approach.

### New Molecular Targets Under Clinical Investigation

Currently, three new molecular targets, emerged from biochemical studies, are under investigation as possible pharmacological strategies to treat LMNA-CMP. The first approach involves a selective oral inhibitor of the p38 MAPK pathway is currently under a phase III clinical trial^2^. The second approach regards the application of mTOR pathway inhibitors that have shown promising improvements in terms of LV size and function in animal models (Choi et al., 2012), and the third approach focuses on PDGFR blockers and their ability to ameliorate the arrhythmic phenotype in *in vitro* models (Lee et al., 2019).

## Molecular Hypotheses Behind the “Clinical Scenario”

As mentioned above, 498 different mutations in *LMNA* gene have been reported, of which 165 associated with LMNA-CMP. Since the discovery of the *LMNA* gene as causative of laminopathies, more than 1000 research and clinical studies have been published, aiming to establish a causal correlation between morphological and functional defects of laminopathic cells and the heterogeneous clinical phenotypes of this group of disorders. Although several hypotheses on the potential mechanisms have emerged from these studies, a unique view on the role of Lamin A/C defects in laminopathies is still missing. The same scenario also applies to CMPs due to *LMNA* mutations and the functional role of A-type lamins in the mammalian heart. Studies on animal models either lacking *Lmna* gene or transgenic for specific human *LMNA* variants, together with evidence from human cardiac models generated through iPSC (induced Pluripotent Stem Cell) technology have significantly contributed to increase our knowledge in the field of cardiolaminopathy. Independent investigations have proposed several hypotheses to explain molecular mechanisms underlying Lamin A/C action in the heart and their link to disease traits.

Nikolova et al. (2004) proposed that the softer nuclei’s structure associated to *LMNA* mutations and the resulting morphological abnormalities may be the molecular events at the basis of the Lamin-related cardiac phenotypes. This has been specifically referred to as the “structural hypothesis,” attributing the lamin-associated phenotypes to structural defects. Furthermore, being an integral part of the LINC complex (Linker of Nucleoskeleton and Cytoskeleton), A-type lamins may also contribute in regulating the structural architecture of the contractile tissue, conferring resistance and protection against any mechanical stress (Swift et al., 2013); this view was later referred to as the “mechano-transduction hypothesis.”

Alongside these “structural hypotheses,” a “gene transcription hypothesis” also emerged, starting from previous evidence suggesting a role of the inner nuclear membrane in regulating chromatin organization (and gene expression). A study from Ye and Worman (1996) provided the first piece of evidence of a link between NL and chromatin regulation: the authors finely demonstrated a specific binding between the human chromodomain proteins HP1 and the integral protein of the inner nuclear membrane LBR (Lamin B Receptor) in *Drosophila*, portraying for the first time, the association between heterochromatin and the inner nuclear membrane in eukaryotic cells. These findings added a new function to Lamin A/C proteins, demonstrating that their biological role is not just restricted to the control of nuclear shape and mechano-transduction, but extends to the active regulation of gene transcription. Following this trail, many other studies subsequently showed that several binding factors are able to simultaneously interact with the chromatin and the NL (Cohen et al., 2001; Wilson et al., 2001; Wilson and Foisner, 2010; Cesarini et al., 2015). This gene transcription hypothesis relies on a two-step model, in which the NL regulates transcription factors, either by sequestering them to the nuclear periphery – usually transcriptionally inactive – or by a mechanism that involves activation of specific signaling pathways, such as ERK1/2 (Arimura et al., 2005; Muchir et al., 2007; Chatzifrangkeskou et al., 2018). In addition to this, Lamin A/C is also able to directly bind chromatin, modulating its spatial organization. The interaction between chromatin and A-type lamins occurs at definite genomic regions, defined as lamin-associated domains (LADs) (Pickersgill et al., 2006). Interestingly, there is evidence showing a remodeling of LADs, in particular those tissue-specific (facultative LADs), in presence of Lamin A/C mutations. These rearrangements do not exclusively occur at the nuclear periphery, but also affect expression of genes located at the nuclear interior (Briand and Collas, 2018). For example, repositioning of *T/BRACHYURY* gene from the nuclear periphery toward the nuclear interior has been reported in fibroblasts of patients affected by familial partial lipodystrophy of Dunnigan type 2 (FPLD2) (Briand et al., 2018). Muck et al. (2012) also showed that knockdown of Lamin A/C in human HeLa cells, cause relocation of *CFTR* gene in the nuclear interior, without affecting the position of the neighboring *GASZ* and *CORTBP2* genes. On the contrary, we recently demonstrated a preferential localization of *SCN5A* gene by the nuclear periphery in human iPSC-CMs carrying the p.K219T *LMNA* mutation, resulting in the inhibition of the expression of the gene and thus leading to reduction of sodium currents and impaired cell excitability (Salvarani et al., 2019).

In addition to this, lamin-chromatin interactions may also occur through several binding factors, identified as interacting-mediators between Lamin A/C and chromatin compartments (Kubben et al., 2010); interestingly some of these have been described as essential elements to regulate transcription of key tissue-specific genes, also in the heart (Briand et al., 2018; Salvarani et al., 2019). From this perspective, this mechanistic model of Lamin A/C action can be more accurately defined as “chromatin hypothesis”; importantly, Lamin A/C-driven chromatin regulation has been recently started to be addressed also in the field of cardiolaminopathy by studies from different research groups including ours (Bertero et al., 2019; Mozzetta and Tedesco, 2019; Salvarani et al., 2019). The general view emerging from chromosome conformation capture studies (HiC), a method to study three-dimensional architecture of genomes enabling to distinguish between transcriptionally active (A) and inactive (B) compartments, is that Lamin A/C haploinsufficiency is associated to an increase of intra-chromosomal interactions (meaning interactions between two active compartments – A-A), without interfering with inter-chromosome interactions (with different transcriptional status – A-B) or inducing massive changes in gene transcription (Bertero et al., 2019). According to that, four dimensional genome conformation (referred as spatial assemblies of heterochromatic topological associated domains – TADs, or “TAD cliques”) showed silencing of developmental genes in human adipose-derived stromal cells (ASCs), without reporting any A to B switch (Paulsen et al., 2019). On the other hand, the study from Lee et al. (2019) associated Lamin A/C haploinsufficiency, caused by a different frameshift *LMNA* mutation, with relevant LAD alterations, that are responsible for substantial changes in gene expression profile leading to altered activation of PDGF pathway. Although the “chromatin hypothesis” has been associated so far to few specific variants, it is likely that similar regulatory mechanisms are driven also by other Lamin A/C mutations, reinforcing the link between changes in transcriptional regulation and chromatin organization occurring in Lamin A/C mutant cells and the respective clinical phenotype.

Consistently, chromatin remodeling and the associated transcriptional changes of disease-relevant genes, is also the prevalent model proposed for the HGPS, the most severe *LMNA*-dependent disease (Goldman et al., 2004; Gordon et al., 2014; Aguado et al., 2019). Interestingly, a very recent study by Ikegami et al. (2020) further contributed to the field describing a new regulatory mechanism by Lamin A/C. Specifically, the authors describe a new regulatory function of the nucleoplasmic Ser22-phosphorylated (pS22) Lamin A/C, that they found specifically bound to a subset of active enhancers, and show the acquisition of new pS22-Lamin A/C binding site in HGPS cells, leading to upregulation of clinically relevant genes and potentially underlying the mechanism behind the disease phenotype.

Overall, data obtained so far from different studies in the field support a model by which all the proposed hypotheses can be causally connected, rather than mutually exclusive. As suggested by Osmanagic-Myers and Foisner (2019) in their Perspective, it is likely that a robust link between the structural abnormalities of laminopathic nuclei (Nikolova et al., 2004; Galiová et al., 2008) and either chromatin modifications or reorganization exists, thus contributing to a multifaceted mechanistic model underlying the pathogenesis of the disease.

## Mouse Models

In the past two decades, several mouse models have been generated, either reproducing patients’ mutations (*LMNA* transgenic mice) or studying the consequences of *Lmna* deficiency (*Lmna* knockout mice). *Lmna^–/–^* mouse developed muscular dystrophy, DCM, neuropathy and displayed retarded growth rate, reduced stores of white fat and cardiac arrhythmia (Sullivan et al., 1999). These mice generally die by 8 weeks of age.

The heterozygous (*Lmna*^±^), with 50% of Lamin A/C expression, manifest conduction system diseases, ventricular dilatation later in adult life and generally die by 8 months of age (Chandar et al., 2010). At the molecular level, both the *Lmna* null and the *Lmna* haploinsufficiency mice showed altered desmin pathway and defective force transmission, leaning toward the mechano-transduction hypothesis. Furthermore, those models also showed an increase of pro-adipogenic factors (PPAPγ and CEBP/α), whereas Wnt-10/β-catenin levels were decreased (Tong et al., 2011). Another two *Lmna* knock-out models, *Lmna^*GT*–/–^* and *Lmna^*delK*32/delK32^*, have also contributed significantly to establish the role of Lamin A/C in postnatal maturation, showing developmental impairment and growth retardation, that mostly affect the adipose tissue, the skeletal muscle and the heart (Kubben et al., 2011; Bertrand et al., 2012).

Recently, the generation of *Lmna*^H222P^ and *Lmna*^N195K^ transgenic mice (Arimura et al., 2005; Mounkes et al., 2005) successfully recapitulated a faithful model of skeletal muscle and DCM, with no phenotype at neonatal stage and displaying disease traits similar to patients. The pathogenesis of *Lmna*^H222P^ mice was linked to elevated MAP kinase 1/2 (ERK1/2) and AKT/mTor signaling pathways (Muchir et al., 2007; Chatzifrangkeskou et al., 2016). Antoku et al. (2019) recently further characterized this model, describing a novel relationship between the elevated levels of ERK1/2 and nuclear positioning in cardiomyocytes (CMs) isolated from *Lmna*^H222P/H222P^. The authors showed that a single phosphorylation site in the formin homology domain-containing proteins (FHOD) can be targeted by ERK1/2, changing cell polarity and negatively regulating cell migration. Nuclear positioning is strictly linked to the formation of the sarcomeres during skeletal muscle maturation (Sewry et al., 2001). Consistently, the authors showed that H222P mutation in the *LMNA* gene is associated to altered nuclear positioning and formation and function of sarcomeres, contributing to the pathogenesis of cardiomyopathy. On the other hand, *Lmna*^N195K^ mice displayed an abnormal expression and/or localization of connexin 40 and connexin 43 and sarcomeres disorganization associated to the main DCM phenotype and conduction system defects (Mounkes et al., 2005). A complete list of the described mouse models and their relevant cardiac phenotypes and molecular characteristics are provided in the Table 1.

**TABLE 1:** Mouse models of cardiolaminopathy.

**Mouse model**	**Phenotype**	**Molecular mechanism**	**References**
***Lmna***^–/–^	Muscular dystrophy; DCM; signs of axonal neuropathy; reduction of adipose tissue; death by 8 weeks of age	Structural and Mechano-transduction hypotheses based on abnormal desmin network and defective force transmission	Sullivan et al., 1999; De Sandre-Giovannoli et al., 2002; Nikolova et al., 2004
***Lmna***^+/–^ (Haploinsufficiency)	Phenotypes are less severe than in *Lmna^–/–^*Conduction system defects; DCM; apoptosis of the conduction tissue; death by 8 months of age	Structural and Mechano-transduction hypotheses: – Abnormal desmin network and defective force transmission; – Mechanical-stress induced apoptosis	Wolf et al., 2008; Chandar et al., 2010; Cupesi et al., 2010
		Gene transcription hypothesis: – Reduced expression of *Egr-1* gene due to a direct interaction between Lamin A/C and c-Fos in response to pressure overload	Cupesi et al., 2010
***Lmna***^N195K^ knock-in	DCM-CD	Structural and Signaling hypotheses: – Misexpression/mislocalization of Cx40 and Cx43 – Abnormal desmin organization	Mounkes et al., 2005
***Lmna***^H222P^	Muscular dystrophy and DCM-CD	Structural and signaling hypotheses: – Alteration of ERK/MAPK pathway	Arimura et al., 2005; Muchir et al., 2007; Choi et al., 2012; Muchir et al., 2012; Chatzifrangkeskou et al., 2016; Chatzifrangkeskou et al., 2018; Antoku et al., 2019
		– Link between ERK1/2 and repositioning of cell nuclei	Antoku et al., 2019
***Lmna***^GT^*^–^*^/^*^–^* (*Lmna* null mouse to study cardiac development)	Growth retardation; defects in heart development; decreased amount of subcutaneous fat; death by 2–3 weeks of age	Alteration in gene transcription profile: delayed muscle and cardiac differentiation/maturation.	Kubben et al., 2011
***Lmna***^delK32/delK32^ (*Lmna* null mouse to study cardiac development)	Growth retardation; defects in heart development; decreased amount of subcutaneous fat; death by 2–3 weeks of age	Gene transcription profile defects: deregulation of genes involved in cell metabolism and adipogenesis.	Bertrand et al., 2012

*DCM, dilated cardiomyopathy; DCM-CD, dilated cardiomyopathy-conduction defect; Cx40, Connexin 40; Cx43, Connexin 43.*

To conclude, despite likely species-specific limitations, studies conducted so far using mouse models have been fundamental to identify signaling pathways relevant to development of new therapeutics, such as the previously mentioned p38 inhibitor ARRY-371797 – already in Phase III clinical trial (NCT02057341) – and the N-acetyltransferare 10 (NAT10) inhibitor, Remodelin (Balmus et al., 2018). However, although these are important achievements, there is still an important lack of knowledge on the pathogenetic mechanisms underlying this complex disease and therefore further research in the field is mandatory to comprehensively tackle its molecular basis and develop more specific and effective therapies (Stewart et al., 2007; Zhang et al., 2013).

## Cardiomyocytes From iPscs: a Powerful Platform to Model Human Cardiolaminopathy

The development of iPSC technology has been a groundbreaking revolution in all areas of research, allowing us to generate *in vitro* any cell type of interest, including human CMs, in which pathogenic mechanisms of diseases may be fully investigated (Xu et al., 2002; Moretti et al., 2010; Lodola et al., 2016; Lee J.H. et al., 2017). Research in the cardiolaminopathy field has also benefit of this technology, since use of iPSC-based models allowed us to overcome the major limitations linked to investigation on animal models and non-cardiac primary cells. Indeed, recreating human phenotypes in mice may not be feasible, due to species-specific differences between mice and humans, especially in relation to the cardiovascular system (i.e., electrophysiological properties of cardiac cells) (Davis et al., 2011). On the other hand, obtaining cells from the human heart may be difficult, due to the limited access the organ and the poor survival of the heart cells grown *ex vivo*. For these reasons studies on cardiolaminopathies so far have been mainly conducted on other somatic cell types, such as fibroblasts, skeletal muscle cells and adipocytes, which are easy to access and to maintain *in vitro* (Ho et al., 2011).

Thus far seven independent studies have investigated human LMNA-CMP using iPSCs either derived from patients carrying different *LMNA* mutations or generated through genome editing (Supplementary Table S1). The first set of studies – those published from 2011 to late 2018 – were mainly descriptive, reporting morphological and functional characteristics of cardio-laminopathic cells: these studies thus confirmed the abnormal morphology of cell nuclei and investigated the susceptibility to electrical and mechanical stresses and the alteration of excitation-contraction coupling in contractile cardiac cells (Ho et al., 2011; Siu et al., 2012; Lee Y.K. et al., 2017). The reported phenotypes were generally ascribed to a structural and/or mechano-transduction hypotheses, in which a generated physical force is responsible for the sarcomeres’ disorganization and contribute to alter the NL structure. In a few studies, defects due to *LMNA* mutations were associated to alteration of ERK1/2 signaling pathway, that itself influences the correct assembly of the sarcomeres (Siu et al., 2012; Chatzifrangkeskou et al., 2018).

More recently, similarly to what reported in other cellular models of laminopathy, the pathogenesis of the LMNA-CMP has been linked to the “gene expression/chromatin organization hypothesis,” showing a causal association between *LMNA* mutations, transcription and specific cell phenotypes (Bertero et al., 2019; Lee et al., 2019; Salvarani et al., 2019). More specifically, these studies utilized models of cardiolaminopathy carrying different mutations (i.e., K219T, R225X, and R190W) and found an altered expression of genes of key pathways for CMs functionality. Whether these effects on gene expression are preferentially mediated by remodeling of LADs or the results of a rearrangement at a different level of chromatin organization/regulation (i.e., TADs; change of affinity of mutant Lamin A/C for chromatin binding proteins) still need to be clearly determined.

What is important to highlight, from the functional point of view, is that the robust association between the disease phenotypes and *LMNA* defects was supported by the experiments conducted in isogenic iPSC paired lines, in which the *LMNA* mutations were corrected or inserted through CRISPR/Cas9-based (Bertero et al., 2019; Salvarani et al., 2019) or TALEN (Lee et al., 2019) technologies. Also, these studies provided the identification of new potential therapeutic targets (*SCN5A* gene and PDGF pathway) to treat cardiolaminopathy.

These are important achievements in the field but, with the constant amelioration of the protocols for CM differentiation, we expect to improve even further and to gain more insights into the pathogenetic mechanisms of the disease. Indeed, data available so far come from studies on human CMs obtained from iPSCs mostly through 2D-based differentiation protocols, known to generate CMs with an immature fetal-like phenotype, meaning that they lack some of the structural (i.e., T-tubules, sarcomeric alignment), functional (i.e., calcium handling and contractile properties differences) and metabolic (i.e., glycolysis vs. fatty acid oxidation) properties typical of an adult CM. These properties are highly desirable for drug screening and for modeling specific, adult-onset traits of disease (Karbassi et al., 2020; Mazzola and Di Pasquale, 2020). Given the developmentally regulated expression of Lamin A/C and the complexity of disease phenotype, cell immaturity is likely to impact also on LMNA-CMP modeling, and some important traits of the disease may have been missed so far. The iPSC field is now moving toward the application of 3D-based differentiation protocols, shown to improve maturation of the differentiated CMs, enhancing the potential of iPSC-based cardiac platforms for both disease modeling and drug screening purposes, and from which the cardiolaminopathy field will also benefit in the near future.

## Conclusion

Consensus has yet to be reached on the pathogenetic mechanism of cardiolaminopathy and researchers in the field are still trying to finely dissect the molecular role of A-type lamins in the heart and to establish clear genotype/phenotype correlations. Indeed, cardiolaminopathy is a quite complex diseases, with patients exhibiting extremely variable phenotypes, as regards of either specific clinical manifestations or the severity and progression of the disease.

**FIGURE 1:**
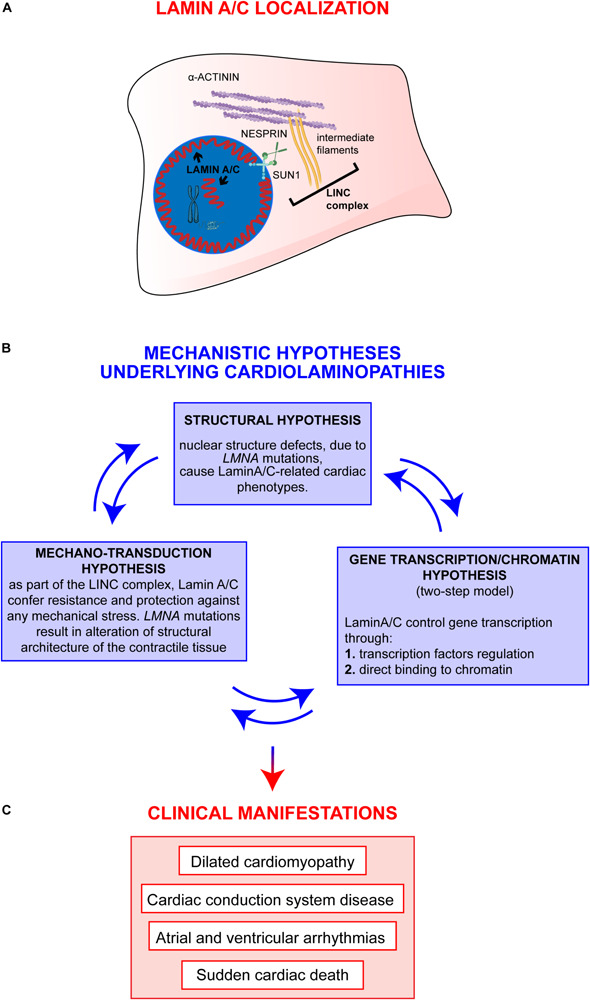
Overview on Lamin A/C proteins localization and on major mechanistic hypotheses underlying cardiac phenotypes. **(A)** Graphic representation of Lamin A/C proteins localization within the cell: Lamin A/C can be found both, at the periphery and in the nuclear interior. In the nucleus, Lamin A/C have a key structural function and are also involved in chromatin organization and regulation of genes transcription. This latter function can be mediated by both peripheral and nucleoplasmic Lamin A/C forms. Besides their role inside the nucleus, Lamin A/C also impact on cellular processes taking place at the outer part of the nuclear envelope: Lamin A/C indeed interact with the nucleo-cytoskeletal proteins, here indicated as LINC complex (i.e., SUN1, NESPRIN, intermediate filaments). The LINC proteins, in turn, interact with other cytoskeletal proteins (i.e., alpha-actinin), contributing to the maintenance of nuclear and cytoskeletal structure and effectors of specific signaling pathways. **(B)** The diagram shows the main mechanistic hypotheses underlying clinical manifestations of cardiolaminopathies. The arrows connecting the blue boxes indicate that these hypotheses are not mutually exclusive, but, instead, are potentially interconnected and all contribute to the final phenotype. **(C)** Clinical manifestations typically associated to LMNA-CMP.

Decades of studies in the field clearly sustain a view in which epigenetic regulations and environmental stimuli are likely to act alongside a single *LMNA* mutation in determining the final cardiolaminopathy phenotype. More recent studies have started to unravel those mechanisms showing (a) the involvement of other “actors” cooperating with a defective Lamin A/C (Salvarani et al., 2019) and (b) the dual impairment of mechano-signaling and gene expression in laminopathies (Osmanagic-Myers and Foisner, 2019), supporting a holistic model in which the different mechanistic hypotheses cooperate with different extents to the final clinical phenotypes observed in patients (Figure 1).

We strongly believe that future studies will further support this global model, unveiling novel disease-relevant pathways and specific targets to treat LMNA-CMP.

## Author Contributions

SC and IM performed the bibliographic search and wrote the manuscript. ED wrote and revised the manuscript, provided funding. All authors contributed to the article and approved the submitted version.

## Conflict of Interest

The authors declare that the research was conducted in the absence of any commercial or financial relationships that could be construed as a potential conflict of interest.

## Footnotes

1 http://www.umd.be/LMNA/

2 www.clinicalTrials.gov identifier NCT03439514
