# Supplementary material for: The Broad Spectrum of LMNA Cardiac Diseases: From Molecular Mechanisms to Clinical Phenotype
Source: Front Physiol. 2020 Jul 3;11:761. doi: 10.3389/fphys.2020.00761 (PMC7349320; doi:10.3389/fphys.2020.00761)
Supplement: Supplementary file 1 [file Table_1.docx]

**The broad spectrum of *LMNA* cardiac diseases: from molecular mechanisms to clinical phenotype**

**Silvia Crasto^1,2§^, Ilaria My^1§^ and Elisa Di Pasquale^1,2*^**

**SUPPLEMENTARY MATERIAL**

**Supplementary Table S1:** iPSC-based models of cardiolaminopathy

| iPSC-derived  cells type | *LMNA*  mutation | Clinical  phenotype | Molecular  mechanism and pharmacological rescue | References |
| --- | --- | --- | --- | --- |
| Fibroblasts | GCCA insertion  (haploinsufficiency) | DCM with mild biventricular dilation (EF 48%); complete AVB | N/A  Authors speculate about the mechanical and gene transcription hypotheses | Ho JC et al, 2011 |
| CMs | R225X  (haploinsufficiency) | Atrial fibrillation, complete AVB, ventricular tachyarrhythmias, ICD implantation; DCM with biventricular dilation (EF 35%) | Structural and signaling hypothesis:  - ERK1/2 pathway alterations  - Accelerated nuclear senescence  - Apoptosis of iPSC‐CMs under electrical stimulation, which can be significantly attenuated by therapeutic blockade of stress‐related ERK1/2 pathway. | Siu CW et al, 2012 |
|  | GCCA insertion  (haploinsufficiency) | See above (Ho JC et al, 2011). |  |  |
| CMs | R225X | R225X: Familiar history of sudden cardiac death, atrial fibrillation, complete AVB, ventricular tachyarrhythmias, ICD implantation; DCM with biventricular dilation (EF 35%). | PTC124 treatment (a small molecule that induces ribosomal readthrough of nonsense mutations)  increased the production of full-length LaminA/C proteins in the R225X mutant only with positive effects on nuclear blebbing, apoptosis and excitation-contraction coupling. | Lee YK et al, 2017 |
|  | Q354X | Atrial fibrillation, complete atrioventricular block, ventricular tachyarrhythmia, ICD implantation and heart failure. | No effect of PTC124 treatment on production of full-length Lamin A/C proteins. |  |
|  | T518fs | Familiar history for atrial fibrillation and complete atrioventricular block. | No effect of PTC124 treatment on production of full-length Lamin A/C proteins. |  |
| CMs | R190W | DCM associated with frequent ventricular ectopic beats | Structural and signaling hypothesis:  - Alterations of ERK/MAPK pathways: active phosphorylated ERK1/2 directly binds to the actin depolymerizing factor cofilin-1 and catalyzes its phosphorylation on Thr25. | Chatzifrangkeskou M et al, 2018 |
| CMs | K219T | Complete AVB, LBBB and ICD implantation, supraventricular and ventricular tachyarrhythmias; severe DCM leading to heart transplantation | Gene transcription/chromatin hypothesis:  - Epigenetic modulation:  K219T-Lamin A/C cooperates with Polycomb Repressive Complex 2 in downregulating *SCN5A* gene expression, leading to decreased sodium current density and slower conduction velocity. | Salvarani N et al, 2019 |
| CMs | R225X (c.673C>T)  (haploinsufficiency); | Severe conduction disease; DCM | Gene transcription/chromatin hypothesis:  - Epigenetic modulation.  Large-scale changes in chromosomal topology: differences in chromatin compartmentalization limited to a few hotspots that escape segregation to the nuclear lamina and inactivation during cardiogenesis but do not explain most gene expression alterations occurring in mutant iPSC-CMs. | Bertero A et al, 2019 |
| CMs | 348-349insG; K117fs  (haploinsufficiency) | Atrial fibrillation and atrioventricular block, ventricular tachycardia;  DCM | Gene transcription hypothesis:  - PDGF signaling activation. Pharmacological and molecular inhibition ameliorated the arrhythmic phenotypes *in vitro.* | Lee J et al, 2019 |

CMs: cardiomyocytes; DCM: dilated cardiomyopathy; EF: ejection fraction; AVB: atrio-ventricular block; ICD: implantable cardioverter defibrillator; LBBB: xxxxx;
